# Putting newborn hearing screening on the political agenda in Belgium: local initiatives toward a community programme – a qualitative study

**DOI:** 10.1186/1478-4505-12-32

**Published:** 2014-07-01

**Authors:** Bénédicte Vos, Raphaël Lagasse, Alain Levêque

**Affiliations:** 1 Université libre de Bruxelles, School of Public Health, Research Center Health Policy and Systems – International Health, Route de Lennik 808, 1070 Brussels, Belgium; 2 Centre d’Epidémiologie Périnatale (CEpiP), Route de Lennik 808, 1070 Brussels, Belgium; 3 Université libre de Bruxelles, School of Public Health, Research Center Epidemiology, Biostatistic and Clinical Research, Route de Lennik 808, 1070 Brussels, Belgium

**Keywords:** Agenda-setting, Health policy, Newborn hearing screening, Policy making

## Abstract

**Background:**

The Kingdon model, based on the convergence of three streams (problem, policy, and politics) and the opening of a policy window, analyses the process by which a health issue is placed on the political agenda. We used this model to document the political agenda-setting process of the newborn hearing screening programme in Belgium.

**Methods:**

A qualitative study based on a document review and on semi-directed interviews was carried out. The interviews were conducted with nine people who had played a role in putting the issue in question on the political agenda, and the documents reviewed included scientific literature and internal reports and publications from the newborn hearing screening programme. The thematic analysis of the data collected was carried out on the basis of the Kingdon model’s three streams.

**Results:**

The political agenda-setting of this screening programme was based on many factors. The problem stream included factors external to the context under study, such as the technological developments and the contribution of the scientific literature which led to the recommendation to provide newborn hearing screening. The two other streams (policy and politics) covered factors internal to the Belgian context. The fact that it was locally feasible with financial support, the network of doctors convinced of the need for newborn hearing screening, the drafting of various proposals, and the search for financing were all part of the policy stream. The Belgian political context and the policy opportunities concerning preventive medicine were identified as significant factors in the third stream. When these three streams converged, a policy window opened, allowing newborn hearing screening onto the political agenda and enabling the policy decision for its introduction.

**Conclusions:**

The advantage of applying the Kingdon model in our approach was the ability to demonstrate the political agenda-setting process, using the three streams. This made it possible to identify the many factors involved in the process. However, the roles of the stakeholders and of the context were somewhat inexplicit in this model.

## Background

In public health, political agenda-setting and policy decision-making are analysed using various concepts and models. In many cases, this type of analysis tends to present what happened, but not how it happened [1]. It often considers that there is some linearity between political agenda-setting, policy formulation, and its implementation and evaluation, implying that there is a clear demarcation between these stages, which in reality is not the case [2,3]. Moreover, these approaches often present health policy analysis models that focus on content, but which overlook the stakeholders, the context, and the political process.

A more dynamic model explaining political agenda-setting for a specific health issue was developed by Kingdon [4,5]. This explains how certain problems become sufficiently significant to get on the political agenda while others do not. It also makes it possible to analyse the reasons why certain proposals to address these problems are considered and then transformed into public policies. This model is based on the existence of three independent streams, each subject to their own influences. When there are certain specific couplings, these three streams converge, opening a policy window in which circumstances are favourable for political agenda-setting. Some authors hold that this convergence is random, while others believe that it is more than simply accidental; it can be due, for instance, to actions or factors associated with policy or organisational cycles. Conversely, these three streams may uncouple, thus temporarily or permanently closing the policy window [1,3-5].

The first stream is the problem stream [3-5]: problems may be brought to the attention of decision-makers in various ways, by monitoring indicators or existing policies, by interest groups (such as medical profession), by the media, or by specific events. The problem must be perceived as a public issue requiring action by the public authorities. The second stream (policy stream) [3-5] is made up of proposals or alternatives developed to address this problem. In this stream, a range of proposals are explored, the majority of which will not come to anything. To be considered, proposals must meet certain criteria concerning equity, technical feasibility, and congruence with society’s values; they must also anticipate future feasibility constraints such as human resources and financing. The third stream (politics stream) [3-5] is made up of ‘events’: it develops independently of the first two streams. These events may be government changes or actions by interest groups. When the three streams converge, a problem is recognised as such, a solution is available, and the political climate means that the time is right for change and there are no constraints to prevent the action [4,5].

In the field of preventive medicine, the introduction of some systematic screening programmes has been performed by government decision. Whatever the target population, this type of screening involves looking for asymptomatic conditions using standard, systematic testing with the aim of identifying people likely to be suffering from the condition so that they can be referred for definitive diagnosis [6]. When screening is applied to infants, it aims to detect serious diseases in asymptomatic newborns as early as possible in order to effectively initiate treatment that might prevent the disease’s future development or, failing that, appropriate care [7].

With the aim of early detection, screening newborns for certain metabolic diseases has been recommended for decades [8]. In Belgium, congenital diseases such as phenylketonuria and galactosemia have been screened for at birth for more than 30 years, following decisions made by the public authorities to provide this screening [9]. More recently, universal hearing screening during the neonatal period has also been recommended by groups of European and North American experts [10,11]. Despite these recommendations, hearing impairment is not systematically screened for during the neonatal period in certain countries. In Belgium, the Wallonia-Brussels Federation (FWB; *Fédération Wallonie-Bruxelles*), formerly known as the French Community, officially launched its newborn hearing screening programme at the end of 2006, while the Flemish Community decided in 1998 to offer screening via its Mother and Child Welfare Agency [12].

The aim of this study was to document the political agenda-setting process that led to the introduction of the newborn hearing screening programme in the FWB. The context for the study was the FWB, which exercises its authority over the French-speaking citizens of Belgium.

## Methods

This was a qualitative study, based on a document review and on semi-directed interviews carried out with participants identified as having contributed to the political agenda-setting and introduction of the newborn hearing screening programme in the FWB.

The participants were from the medical and political communities, from healthcare organisation services, or were patient representatives: they were ENT specialists working in university (n = 3) and non-university (n = 1) hospitals, a paediatrician from the *Office de la Naissance et de l’Enfance* (ONE – the FWB’s Mother and Child Welfare Agency), a health insurance fund representative, and two representatives of the minister responsible for health prevention policy at the time of the decision to introduce the newborn hearing screening programme. These interviews took place between February and September 2012, after which an association of parents of hearing-impaired children, representing the patients’ point of view, was also contacted and included in the study. Thus, a purposeful sampling was used and no criterion of redundancy was defined.

The interview guide, with open-ended questions, was comprised of three parts. The first aimed to collect information on existing hearing screening activities in the FWB prior to the introduction of the newborn hearing screening programme and to identify any synergies between the stakeholders involved, the various actions carried out to implement the programme, and the context associated with this issue. The second part focused on the consultation process and the drafting of the programme’s organisation protocol, and the third aimed to understand the choices made in the drafting of the programme’s organisation protocol. The information collected in the first part of the guide was used in this analysis. It was supplemented by documents (letters, minutes of meetings, working documents, published and unpublished reports) identified during the review of the archives of the programme’s reference centre or during the semi-directed interviews. Scientific literature relating to hearing screening programmes and to hearing impairment in newborns was also reviewed.

The methodological approach used followed the analysis process explained by Varvasovszky et al. [13]. First, we clarified the objectives, the issue of the context, and the level considered for the analysis. We then identified the parties involved and prepared the data collection with, in particular, the creation of an interview guide, which was then implemented. We organised the collected data into diagram format, adding information throughout the collection process. This provided a visual representation of the various factors involved in the political agenda-setting and decision-making process, and revealed the connections between these factors. We used the political agenda-setting theory framework developed by Kingdon to analyse and present the results. The thematic analysis of the interviews and documents examined the issue of a newborn hearing screening programme in the FWB on the basis of the three streams of the Kingdon model. The role of the stakeholders and the context was also explained.

## Results

### Analysis of the problem stream

**Figure 1:**
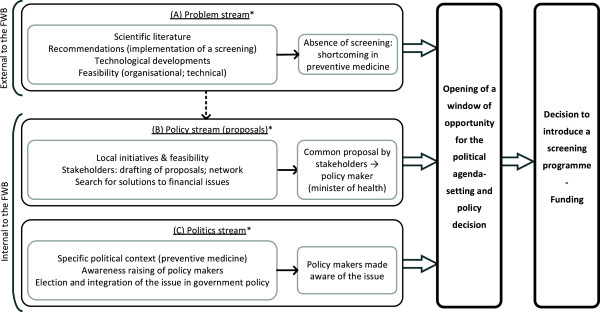
**Application of the Kingdon model (political agenda-setting) to newborn hearing screening in Belgium.** Legend: *Flowcharts of the main steps in the problem stream, the policy stream and the politics stream are presented, respectively, in Figures 2, 3, and 4. FWB: *Fédération Wallonie-Bruxelles* (Wallonia-Brussels Federation).

**Figure 2:**
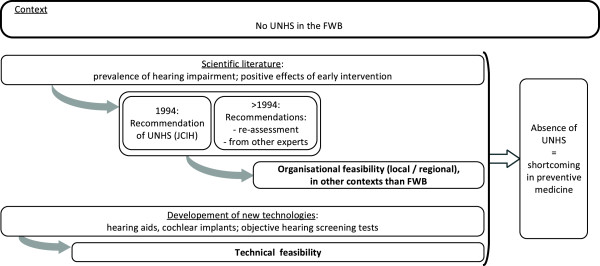
**Flowchart of the main steps in the problem stream (Kingdon model), applied to the newborn hearing screening in Belgium.** Legend: JCIH: Joint Committee on Infant Hearing; FWB: *Fédération Wallonie-Bruxelles* (Wallonia-Brussels Federation); UNHS: Universal Newborn Hearing Screening.

The problem stream centred around the fact that there was no universal hearing screening programme in the FWB, the aim of which is the early treatment of hearing-impaired children in order to allow them to develop to their full potential. Two key factors contributed to this first stream: the scientific literature and technological developments (Figure 1, point A and Figure 2).

According to prevalence data found in the scientific literature, neonatal hearing impairment is a frequent condition: at least 1‰ of newborns suffer bilateral permanent childhood hearing impairment (≥40 dB) [10]. However, it has been demonstrated that providing intervention services for these children in the first months of their life has positive effects on their psycho-social development and language [11,14], while late treatment will have, in particular, a long-term impact (a lower level of education and rate of employment) [11].

On the basis of scientific studies and their expertise, North American and European professional groups have recommended, since 1994, the early diagnosis and treatment of hearing-impaired children along with universal hearing screening in newborns [10,15,16]. All of the recommendations supported the provision of early hearing screening, with the exception of the US Preventive Services Task Force which reported, in 2001, ‘insufficient’ evidence to recommend for or against universal screening in maternity units; in 2008, this decision was reassessed and the level of proof deemed ‘moderate’ [17,18]. In addition, hearing screening had been introduced in certain countries and regions, which demonstrated the organisational feasibility of this type of programme at national or regional level [19-22]. These scientific studies and expert recommendations were used in the FWB in order to champion the provision of early and universal hearing screening, and the professional experience of the doctors interviewed backed this conviction.

This knowledge in the field of newborn and infant hearing (from the benefit of screening to the positive effects of treatment) has come about thanks to the development of new technologies. Hearing devices (hearing aids and cochlear implants) have greatly evolved in recent decades, giving hearing-impaired children more effective hearing systems. Since the 1990s, it has also been possible to screen children from birth using objective, reliable, painless, and rapid methods [23]. These methods use two types of tests (otoacoustic emissions or auditory evoked potentials) and can be applied broadly in screening as the analysis of the tests is automated by a statistical algorithm: the results are presented in binary form (‘good’ or ‘to be monitored’), which means that staff not trained in audiology can carry out the screening. Screening for hearing impairment in newborns is therefore feasible in organisational and technical terms.

This problem stream developed over several decades. It did not include an exceptional event that might suddenly and unexpectedly have brought the issue of the absence of a newborn hearing screening programme in the FWB to the attention of policy makers. However, in light of the scientific and technological arguments, the absence of a newborn hearing screening programme was gradually identified as a shortcoming in preventive medicine in the FWB.

### Analysis of the policy stream

**Figure 3:**
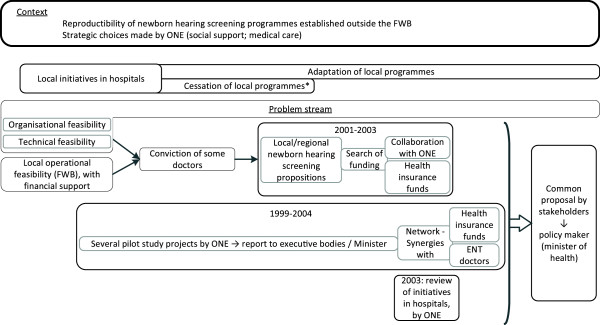
**Flowchart of the main steps in the policy stream (Kingdon model), applied to the newborn hearing screening in Belgium.** Legend: *financial difficulties (mostly). FWB: *Fédération Wallonie-Bruxelles* (Wallonia-Brussels Federation); ONE: Mother and Child Welfare Agency.

This second stream falls within a context in which newborn hearing screening had been carried out for several decades in certain hospitals in the FWB. This consisted of local hospital initiatives or partnerships with intervention services for hearing-impaired children which delegated staff to carry out hearing tests in maternity units. Hearing screening was most often carried out using behavioural tests for which false positives and false negatives were high. Some of these screening programmes had been adapted, while others were terminated for organisational reasons or due to a lack of finances, especially as the Federal Sickness Fund had intervened in order to reduce the extra costs generated by these hearing tests. In 2003, ONE carried out a review of the local newborn hearing screening initiatives in the FWB and found that the protocols applied when screening was provided in maternity units varied. This diversity was apparent both in the methods (screening that is carried out at the request of the parents [non-systematic], targeted at newborns at risk of hearing impairment, or universal in certain hospitals), and in the choice of hearing tests used (objective methods or behavioural tests). Where hospitals were not offering hearing tests, reasons for the non-provision or termination of a hearing screening programme were mainly linked with the lack of reimbursement for the screening process and therefore financial difficulties in providing the screening. This situation showed that, in the local context specific to these hospitals and despite the diversity of the protocols, newborn hearing screening was feasible in the FWB if the financial conditions there were favourable (Figure 1, point B and Figure 3).

Given the arguments developed in the problem stream and the local operational feasibility, hospital doctors in the FWB were convinced of the need to provide newborn hearing screening locally or at a regional level. On a practical level, they identified the advantages and limitations of the various screening methods and modes of organisation in the newborn hearing screening protocols applied in other regions or countries, in order to apply them in the FWB. They developed several local or regional newborn hearing screening programme proposals and, between 2001 and 2003, they sought funding from health insurance funds or through cooperation with ONE.

In addition to this, ONE demonstrated its interest in newborn hearing screening via its paediatric consultant committee. Between 1999 and 2004, the paediatric consultants prepared several pilot study projects for ONE to carry out newborn hearing screening, but none of them were implemented. As these projects were being developed, synergies developed, giving rise to proposals. Work alongside hospital ENT specialists and a health insurance fund gradually guided the proposals to make them more operational for the context in question, particularly the financial context. Initial proposals considered screening by ONE staff during ONE consultations. In order to reduce costs (training and the equipment required), it was proposed that the hearing tests be carried out by ONE workers in maternity units, and it was then proposed that the newborn hearing screening be carried out in maternity units by hospital staff and that ONE coordinate the programme. The latter eliminated one of the problems raised by the previous proposals, as part of ONE workers in contact with newborns were not nurses but social workers, unqualified to carry out hearing tests. The proposals by ONE’s paediatric consultants were reported on several occasions to the executive bodies and the minister concerned, but it seems that the strategic decisions made for ONE by these bodies did not include the introduction of newborn hearing screening within the institution at that time.

In order to overcome the financial barriers, health insurance funds were contacted and federal financing was considered, but these proposals were not taken up.

In the development of the proposals, the reproducibility of the newborn hearing screening programmes established outside of the FWB was studied. The favoured example was the programme implemented in the Dutch-speaking part of Belgium (Flanders) since 1998, in which the newborn hearing screening programme is carried out by the Mother and Child Welfare Agency [12]. Hearing tests are carried out using an objective method at four weeks and at the child’s home. The provision of this newborn hearing screening could not be transposed to the FWB context for two primary reasons. The first concerned the strategic choices by ONE, whose work is more focused on social support than medical care (as demonstrated by the profile of ONE’s workers, who are mostly social workers). The second is a quantitative observation: the Flemish Mother and Child Welfare Agency has a higher rate of coverage of children followed-up by the institution than the French-speaking system [12,24].

In this second stream, the stakeholders supporting the proposals had a specific role: these doctors, who were not acting on behalf of a medical association, became involved in the project on an individual level. They did not have expert knowledge of political lobbying or the use of conclusive data for policy decision-making and do not seem to have used these tools to argue for the adoption of their proposals. The arguments used to promote the proposals were essentially scientific and feasibility arguments, and the proposals developed were only sent to policy makers after a long delay.

Analysis of this policy stream revealed the following: i) the provision of newborn hearing screening was locally possible in maternity units in the FWB, with financial support; ii) a network of professionals (ONE, hospital doctors, health insurance funds) convinced of the need to provide newborn hearing screening had developed and had prepared several newborn hearing screening protocols, adapted to the context of the FWB; iii) ONE, which works in the field of preventive medicine and early childhood, had participated in the drafting of the newborn hearing screening proposals and played a major role in some of the proposals prepared; and iv) at the beginning of the 2000s, the lack of financial resources seems to have been the main obstacle to introducing a newborn hearing screening programme based on proposals from both hospital doctors and ONE’s paediatric consultants.

### Analysis of the politics stream

**Figure 4:**
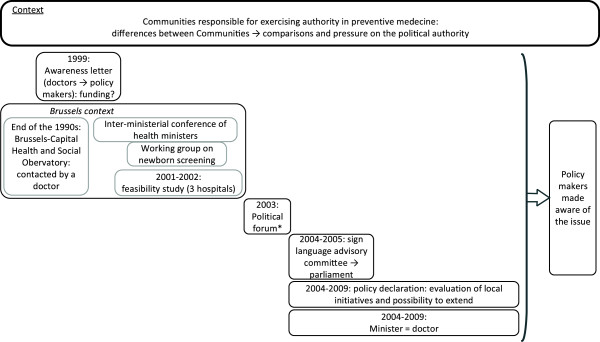
**Flowchart of the main steps in the politics stream (Kingdon model), applied to the newborn hearing screening in Belgium.** Legend: *organised by an association of parents of hearing-impaired children.

Raising the awareness of policy makers on the issue of newborn hearing screening was introduced at the end of the 1990s. In 1999, the attention of policy makers was drawn to the need to provide newborn hearing screening via an awareness letter jointly signed by doctors requesting the funding for this screening. During this same period, but in the Brussels context, an ENT doctor approached the Brussels-Capital Health and Social Observatory to obtain backing to introduce newborn hearing screening in the region. This did not take place due to a lack of financial resources and the fear that some Brussels’ inhabitants would not buy into the screening, which would undermine its scientific relevance if it were not systematic and universal. Following this move, a feasibility study was carried out in 2001–2002, in three Brussels hospitals. This study concluded that providing newborn hearing screening was feasible if certain conditions were met, such as taking into account the complexities of the health system and the fragmentation of healthcare activities. In addition, as a result of an inter-ministerial conference of health ministers, a working group on newborn screening was created, but it was stopped at the beginning of the 2000s with no specific progress being made on the issue of newborn hearing screening (Figure 1, point C and Figure 4).

Other stakeholders also raised awareness among policy makers on the need to introduce newborn hearing screening: in 2003, an association of parents of hearing-impaired children organised a political forum, and in 2004 and 2005 a working group from the FWB’s sign language advisory committee addressed members of parliament on the need to provide newborn hearing screening.

In 2004, elections were held and the policy declaration of the FWB government for the 2004 to 2009 period planned to evaluate existing local newborn hearing screening projects in maternity units and to study the possibility of extending these across the FWB. During this period, ministerial authority in preventive medicine and health promotion was held by a doctor, an uncommon situation in Belgium.

In this third stream, the role of the levels of power responsible for exercising authority in preventive medicine should not be overlooked. In the Belgian health system, the federal level manages issues related to curative medicine while the communities are responsible for preventive medicine and health promotion. The Communities (French- [that is, the FWB], Flemish-, and German-speaking) therefore have the opportunity to develop their own prevention policies, giving rise to a platform for comparison between the screening programmes available to the populations of the Communities. As regards newborn hearing screening, the Flemish Community has had a programme since 1998, which was not the case in the FWB, meaning that Belgian newborns had unequal chances of receiving a hearing test and early treatment in the event of hearing impairment depending on the Community (French or Flemish) to which they belonged. This discrepancy gave rise to political pressure for the FWB to catch up in the field of newborn hearing screening.

### Coupling of the three streams and opening of a policy window

At a particular moment, these three streams converged. The provision of newborn hearing screening was recognised as necessary to allow hearing-impaired children to develop to their full potential through early treatment. Technological developments had made it possible to effectively treat hearing-impaired children and carry out reliable hearing tests from birth. Local experiences in the FWB, and national and regional experiences outside the FWB, supported the feasibility of a newborn hearing screening programme. Various newborn hearing screening proposals had been issued with the aim of developing a programme in the FWB. These proposals had come from hospital doctors and ONE paediatric consultants but had not been put into practice, primarily for financial reasons. Lastly, policy makers were aware of preventive medicine issues and of newborn hearing screening, and when a new government came in in 2004, it was in a context of policy opportunities as regards preventive medicine.

At the end of 2004, these different streams converged. A policy and possible decision window was opened when a newborn hearing screening proposal in partnership with ONE was submitted to the minister to request the funding required for its implementation.

The policy decision by the minister responsible for prevention policy to introduce a newborn hearing screening programme in the FWB led to the creation of a working group. This group was responsible for preparing the protocol for the programme and was composed primarily of the ENT specialists, paediatricians (from ONE), and representatives of health insurance funds who had prepared the various proposals for providing newborn hearing screening. Financial resources were made available to subsidise this programme. In November 2006, the newborn hearing screening programme in the FWB was officially presented and proposed to hospitals: newborn hearing screening would be provided by hospitals with a maternity unit in order to achieve a high rate of coverage and to avoid a high proportion of newborns being lost to follow-up; the participation of the hospitals was voluntary. The programme would be coordinated, from the start, by a university team, with ONE’s role confined to raising parents’ awareness, particularly in the event of the absence of hearing tests or in the presence of non-conclusive results.

## Discussion

The political agenda-setting and policy decision to provide a newborn hearing screening programme in the FWB were based on many factors. Some factors did not concern the context of the FWB, such as the data from scientific literature and the technological developments that were identified in the first stream, while other factors were specific to the context of the FWB: local operational feasibility, the newborn hearing screening proposals, the synergies between stakeholders, and the political context, all of which were analysed in the second and third streams.

The factors external to the context of the FWB (problem stream) were based on studies and data from the literature. The evidence utilisation is put forward by various authors as a decision support component [2,25,26]. This implies, on the one hand, the availability of data from scientific research which can be used by decision-makers and, on the other, the integration of this data within the context in which the decision is made [26]. Until the end of the 1990s, few evidence-based studies were available on the positive effects of universal newborn hearing screening [17]. However, experts had recommended newborn hearing screening in maternity units and studies showed the relevance of introducing this programme, particularly by taking into account the high rate of coverage or demonstrating the lower age at which patients are diagnosed with hearing impairments. These studies were mostly conducted at country or regional level in contexts other than the FWB, while local experiences within the FWB demonstrated the operational feasibility in this context. This data served as a basis to the argument for the need to introduce newborn hearing screening programme and was transposed to the FWB context in the drafting of newborn hearing screening proposals.

One of the factors specific to the FWB context that contributed to the agenda-setting and decision for newborn hearing screening was the key role of the stakeholders in addressing policy makers; this ties in with Exworthy’s findings [3]. In our analysis, paediatric consultants (from ONE), through their executive powers, addressed policy makers and the minister concerned on the need to provide and finance newborn hearing screening. However, the hospital doctors, who had also issued proposals, did not approach the government authorities and the search for funding was initially focused on other bodies. These stakeholders, clinical practitioners, are not accustomed to using lobbying or decision support tools. Yet, the lack of financial resources was identified as the biggest barrier to implementing the newborn hearing screening proposals; convincing the policy makers responsible for allocating resources was, therefore, essential. In addition, among the potential contributors, it seems that the non-health interest groups, such as associations of parents of hearing-impaired children, played a moderate role in placing the issue on the political agenda. These various groups of contributors coordinated their actions very little, if at all.

In our analysis, the three streams evolved independently and reached maturity at a similar time, although the problem stream had been available before the others as its content fuelled the policy stream. The factors in our study are in line with those developed by Almeida et al., who stated that the transformation of technical and scientific proposals into policy decisions to implement involve much more than the willingness of the stakeholders or the technical quality of the scientific information that recommend such a change. Ideological, political, and economic factors are decisive in the formulation of proposals and the progress of the initiatives decided upon [2]. Indeed, before the policy window opened, various obstacles to the agenda-setting and decision were identified in our study, and the financial constraints were identified as preventing the adoption of the proposals previously formulated. The decision to introduce newborn hearing screening was made because the government authority concerned deemed it relevant to allocate resources to the issue, despite a context of limited financial resources. The reasons behind this decision to allocate finances may vary: policy decisions are rarely made in public and are not systematically the result of a rational process as the context is often highly political [27]. Our study did not investigate this particular point, nor did it analyse the possible impact of the political benefits or electoral stakes of this decision or the role of the coincidence in this process.

The use of the Kingdon model demonstrates the importance of the process that leads to a decision; each stream evolves at its own pace, which cannot be constant or linear. This analysis also identified overlaps between the factors contributing to the political agenda-setting, in which decision-making is a part of the process [3]. However, the model does not explicitly provide for analysis of the context which influences each of the streams, or of the role of the stakeholders. Categorisation into distinct streams also suggests a clear demarcation between them, whereas the reality is much more complex, with possible interactions between the streams.

The collection of data dating back several years was a limitation of this study. The events that occurred may be re-interpreted retrospectively and in the light of the final decision to implement the screening. In order to limit recall bias, we favoured the collection of objective information from documents. Moreover, in addition to the scientific, political, and context factors presented, it is possible that personnel or policy strategy factors may have affected the decision-making process and that this has not been documented in the study. The exhaustiveness of the information collected is difficult to prove, the decision-making process can be unclear, and access to certain documents problematic [1]. The selection of the key-informant participants may also be biased, due to their active role in the implementation of the newborn hearing screening programme, and some other stakeholders, whose action could be considered as less decisive in the decision-making process, not being interviewed; this would result in a lack of completeness in the study.

The position of the researcher and their impact on the research process are also significant in this type of analysis, but they are rarely explained in policy analysis literature. There are two types of researcher position based on whether the researcher participated in the policy process (insider) or otherwise (outsider) and the results may vary between these two approaches [1]. In this study, the researchers did not participate in the political agenda-setting or decision-making process, giving them an outside view in the analysis. However, the researchers did carry out the implementation and evaluation of the screening programme, giving them a thorough knowledge of the programme and also of the stakeholders, which facilitated access to the documents and the people to be interviewed.

## Conclusions

By applying the Kingdon model, our work made it possible to document the political agenda-setting process that led to the introduction of a newborn hearing screening programme in the FWB. Some factors were identified as not concerning the FWB while others resulted directly from the FWB and were related to the context and situation studied. The combination of all of these factors created a context conducive to the political agenda-setting and policy decision, in a process of evidence-informed health policy-making.

## Abbreviations

FWB: *Fédération Wallonie-Bruxelles* (Wallonia-Brussels Federation); ONE: *Office de la Naissance et de l’Enfance* (Mother and Child Welfare Agency).

## Competing interests

The authors declare that they have no competing interests.

## Authors’ contributions

BV participated in the design of the study, collected the data, performed the qualitative analysis, and drafted the manuscript. RL and AL participated in the design of the study, in the redaction of the manuscript, and critically reviewed the text. All authors read and approved the final manuscript.

## Authors’ information

BV has coordinated the newborn hearing screening programme in the FWB since January 2007, RL was the scientific promoter of the programme between September 2006 and November 2012, and AL has been the scientific promoter of the programme since November 2012.
